# Wood’s Household Magazine

**Published:** 1873-09

**Authors:** 


*Wood's Household Magazine.*

The October number of this excellent magazine has thrust its cheery little self into our presence. It brings to us the treasures it has been gathering the past month, and all are deserving of notice. Each has its own peculiar value, and "Maggie" holds up this, that and the other literary gem, and archly *seems* to say, "What do you think of this?" and "Isn't this nice?" and "Look at that?" And we glance at them all and say, "Yes! Yes! *All* are good!" There is also a pretty engraving of the chromo Yosemite, which the publisher is offering as a premium—which it would be well for all our readers to examine—as it gives a very accurate idea, save in size and coloring, of what the chromo Yosemite is. Price of the magazine, one dollar a year; with chromo, one dollar and a half. Address, Wood's Household Magazine, Newburgh, New York.

